# Supplementary material for: Late Evening Snack with Branched-Chain Amino Acids Supplementation Improves Survival in Patients with Cirrhosis
Source: J Clin Med. 2020 Apr 3;9(4):1013. doi: 10.3390/jcm9041013 (PMC7230335; doi:10.3390/jcm9041013)
Supplement: Supplementary file 1 [file jcm-09-01013-s001.pdf]

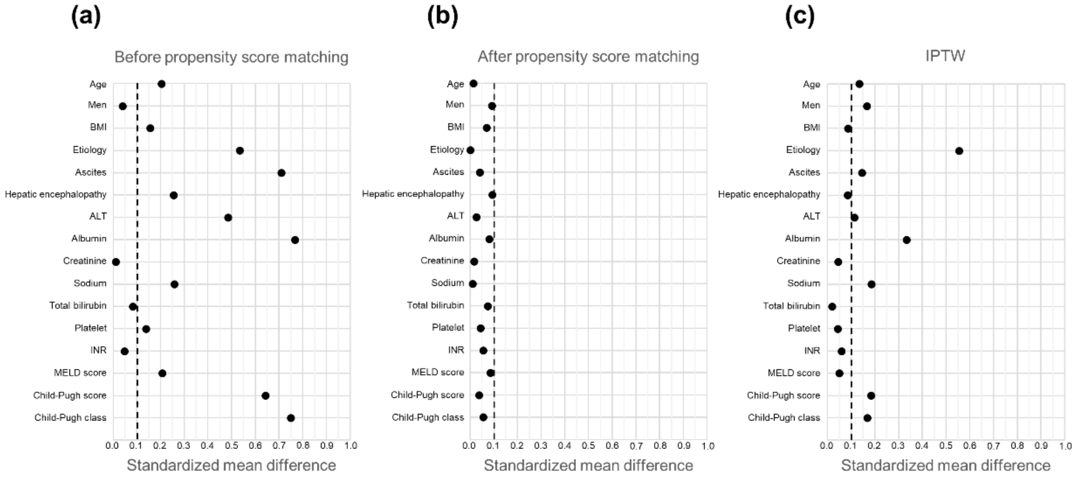

Supplementary Figure S1. Standardized mean difference used to compare the balance between the LES and No LES groups. (**a**) Before propensity score matching analysis. (**b**) After propensity score matching analysis. (**c**) IPTW. ALT, alanine aminotransferase; BMI, body mass index; INR, international normalized ratio; IPTW, inverse probability of treatment weighting; LES, late evening snack; MELD, model for end-stage liver disease.

Supplementary Table S1. Causes of death before and after propensity score matching analysis.

	**Before matching**			After matching		
	**All patients**	**No LES†**	**LES†**	Matched patients	No LES†	LES†
Cause of death	(n = 163)	(n = 142)	(n = 21)	(n = 92)	(n = 72)	(n = 20)
Liver failure	66.3% (108/163)	67.6% (96/142)	57.1% (12/21)	69.6% (64/92)	72.2% (52/72)	60.0% (12/20)
Hepatocellular carcinoma	14.1% (23/163)	14.1% (20/142)	14.3% (3/21)	10.9% (10/92)	11.1% (8/72)	10.0% (2/20)
Infection	3.7% (6/163)	2.8% (4/142)	9.5% (2/21)	3.3% (3/92)	1.4% (1/72)	10.0% (2/20)
Variceal hemorrhage	1.2% (2/163)	1.4% (2/142)	0	2.2% (2/92)	2.8% (2/72)	0
Colon cancer	1.8% (3/163)	2.1% (3/142)	0	1.1% (1/92)	1.4% (1/72)	0
Esophageal cancer	0.6% (1/163)	0.7% (1/142)	0	0	0	0
Laryngeal cancer	0.6% (1/163)	0.7% (1/142)	0	0	0	0
Lung cancer	0.6% (1/163)	0.7% (1/142)	0	1.1% (1/92)	1.4% (1/72)	0
Pancreatic cancer	0.6% (1/163)	0.7% (1/142)	0	0	0	0
Cardiovascular disease	2.5% (4/163)	1.4% (2/142)	9.5% (2/21)	2.2% (2/92)	0	10.0% (2/20)
Interstitial pneumonia	1.2% (2/163)	1.4% (2/142)	0	1.1% (1/92)	1.4% (1/72)	0
Trauma	1.2% (2/163)	1.4% (2/142)	0	1.1% (1/92)	1.4% (1/72)	0

Cerebral hemorrhage	0.6% (1/163)	0.7% (1/142)	0	0	0	0
Myelofibrosis	0.6% (1/163)	0.7% (1/142)	0	1.1% (1/92)	1.4% (1/72)	0
Strangulated ileus	0.6% (1/163)	0.7% (1/142)	0	1.1% (1/92)	1.4% (1/72)	0
Unknown	3.7% (6/163)	2.8% (4/142)	9.5% (2/21)	5.4% (5/92)	4.2% (3/72)	10.0% (2/20)

Values are presented as numbers (percentages). LES, late evening snack. †LES was defined as daily use of a branched-chain amino acid-enriched powder mix before bedtime. Less frequent use or never-use was defined as No LES.

Supplementary Table S2. Variables of the LES and No LES groups after inverse probability of treatment weighting.

Characteristic	No LES† (n = 526)	LES† (n = 470)	*P*-value*	SMD‡
Age (years)	67.0 (56.0–74.0)	68.7 (59.0–73.0)	0.589	0.134
Men	289 (55.1)	297 (63.2)	0.217	0.166
BMI (kg/m²)	22.6 (20.9–25.2)	22.8 (21.1–24.5)	0.776	0.086
Etiology of cirrhosis				
HBV	30 (5.6)	38 (8.0)	0.001	0.555
HCV	243 (46.2)	155 (33.0)		
ALD	94 (17.9)	191 (40.6)		
Others	159 (30.2)	86 (18.4)		
Ascites	212 (40.4)	224 (47.6)	0.295	0.146
Hepatic encephalopathy	42 (8.0)	49 (10.4)	0.461	0.085
ALT (IU/L)	32 (20–49)	30 (17–52)	0.589	0.114
Albumin (g/dL)	3.4 (2.8–4.0)	3.3 (2.8–3.4)	0.008	0.334
Creatinine (mg/dL)	0.72 (0.57–0.89)	0.67 (0.58–0.89)	0.695	0.045
Sodium (mEq/L)	139 (137–141)	138 (136–140)	0.021	0.185
Total bilirubin (mg/dL)	1.10 (0.80–1.63)	1.00 (0.80–1.71)	0.997	0.019
Platelet (10^9/L)	102 (69–157)	92 (60–170)	0.335	0.043
INR	1.09 (1.01–1.23)	1.07 (1.02–1.23)	0.973	0.060
MELD score	9 (7–11)	9 (7–12)	0.714	0.050
Child–Pugh score	6 (5–8)	7 (6–9)	0.011	0.184
Child–Pugh class				
A	291 (55.4)	223 (47.3)	0.421	0.167
B	144 (27.4)	159 (33.9)		
C	91 (17.3)	88 (18.8)		

Values are presented as numbers (percentages) or medians (interquartile ranges). ALD, alcohol-related liver disease; ALT, alanine aminotransferase; BMI, body mass index; HBV, hepatitis B virus; HCV, hepatitis C virus; INR, international normalized ratio; LES, late evening snack; MELD, model for end-stage liver disease; SMD, standardized mean differences. †LES was defined as daily use of a branched-chain amino acid-enriched powder mix before bedtime. Less frequent use or never-use was defined as No LES. *The chi-square test for categorical variables or Mann–Whitney *U* test for continuous variables were used to compare the clinical characteristics between the two groups. ‡SMD was used to compare the balance in baseline variables between the two groups.
